# Crossing the Digital Divide in Online Self-Management Support: Analysis of Usage Data From HeLP-Diabetes

**DOI:** 10.2196/10925

**Published:** 2018-12-06

**Authors:** Shoba Poduval, Saddif Ahmed, Louise Marston, Fiona Hamilton, Elizabeth Murray

**Affiliations:** 1 eHealth Unit Department of Primary Care & Population Health University College London London United Kingdom

**Keywords:** type 2 diabetes mellitus, self-management, patient education, internet, digital divide, social class, health literacy, computer literacy

## Abstract

**Background:**

Digital health is increasingly recognized as a cost-effective means to support patient self-care. However, there are concerns about whether the “digital divide,” defined as the gap between those who do and do not make regular use of digital technologies, will lead to increased health inequalities. Access to the internet, computer literacy, motivation to use digital health interventions, and fears about internet security are barriers to use of digital health interventions. Some of these barriers disproportionately affect people of older age, black or minority ethnic background, and low socioeconomic status. HeLP-Diabetes (Healthy Living for People with type 2 Diabetes), a theoretically informed online self-management program for adults with type 2 diabetes, was developed to meet the needs of people from a broad demographic background.

**Objective:**

This study aimed to determine whether there was evidence of a digital divide when HeLP-Diabetes was integrated into routine care. This was achieved by (1) comparing the characteristics of people who registered for the program against the target population (people with type 2 diabetes in inner London), (2) comparing the characteristics of people who registered for the program and used it with those who did not use it, and (3) comparing sections of the website visited by different demographic groups.

**Methods:**

A retrospective analysis of data on the use of HeLP-Diabetes in routine clinical practice in 4 inner London clinical commissioning groups was undertaken. Data were collected from patients who registered for the program as part of routine health services.. Data on gender, age, ethnicity, and educational attainment were collected at registration, and data on webpage visits (user identification number, date, time, and page visited) were collected automatically by software on the server side of the website.

**Results:**

The characteristics of people who registered for the program were found to reflect those of the target population. The mean age was 58.4 years (SD=28.0), over 50.0% were from black and minority ethnic backgrounds, and nearly a third (29.8%) had no qualifications beyond school leaving age. There was no association between demographic characteristics and use of the program, apart from weak evidence of less use by the mixed ethnicity group. There was no evidence of the differential use of the program by any demographic group, apart from weak evidence for people with degrees and school leavers being more likely to use the “Living and working with diabetes” (*P*=.03) and “Treating diabetes” (*P*=.04) sections of the website.

**Conclusions:**

This study is one of the first to provide evidence that a digital health intervention can be integrated into routine health services without widening health inequalities. The relative success of the intervention may be attributed to integration into routine health care, and careful design with extensive user input and consideration of literacy levels. Developers of digital health interventions need to acknowledge barriers to access and use, and collect data on the demographic profile of users, to address inequalities.

## Introduction

### Background

Health systems internationally are struggling with the challenges posed by rising demand and increasing costs of health care due to an aging population, the increase in the prevalence of long-term conditions, and changing patient expectations [1-3]. This challenge has been clearly articulated in the English National Health Service (NHS), with an explicit commitment to improving the quality and efficiency of care delivered by the NHS, within a tightly controlled budget [4]. Two strategies which have been identified are increasing provision of digital health and promoting self-care by patients [5-9]. The expectation is that, where health care can be effectively delivered through digital means, it will be more cost-effective than face-to-face health care delivery, because of the scalability and low marginal costs per additional user of digital health interventions. There is also an expectation that improving patients’ ability to self-care will reduce health care costs and improve health outcomes.

Although there are some data to support both these contentions [4,10-12], there are also anxieties about the extent to which such policies will widen health inequalities [13]. There are concerns about the “digital divide,” defined as the divide between those who do and do not make regular use of digital technologies and the internet [14,15]. Overall, internet use is high in the United Kingdom (UK), with 90% of the population having access in 2017, and one of the most common reasons for using the internet is to access health-related information or services [16]. However, there are still 4 million households without internet access, and those without access are often those who are most in need of health care, including older people, people with disability, and people with lower socioeconomic status (SES) [17]. Data from the Office for National Statistics show that adults aged over 75 years are the lowest users of the internet [16], and the proportion of adults who are recent internet users is lower for disabled people than it is for able-bodied people [18]. Buying and accessing computer equipment is costly, which presents a barrier to access for people with lower SES.

The digital divide is about more than just access. The 2014 Government Digital Inclusion Strategy identified 3 additional challenges, which were (1) not having the skills or capacity to use the internet (computer literacy), (2) not having the motivation to go online, and (3) lack of trust in internet security [19]. Thus the “digital divide” is closely related to general literacy and health literacy. Health literacy has been defined as “the cognitive and social skills which determine the motivation and ability of individuals to gain access to, understand and use information in ways which promote and maintain good health” [20]. People with low health literacy are less able to access and use health information effectively and have poorer health outcomes [21,22].

Similarly, there are concerns that programs which aim to promote self-care by patients, such as the expert patient program, may widen health inequalities as people with higher levels of self-efficacy, and better access to social, economic, and practical resources, may be better able to engage with such programs and adopt the behaviors required for effective self-management [23,24].

In this paper, we present registration and usage data from a digital program designed to support self-management of type 2 diabetes mellitus (T2DM), known as Healthy Living for People with type 2 Diabetes (HeLP-Diabetes). We collected data on age, gender, ethnicity, and educational attainment. Educational attainment was used as a marker of SES, as is common in epidemiological research [25,26], and also digital and health literacy. Low health literacy has been found to be more common among older people, people from black and minority ethnic (BAME) backgrounds, people with lower incomes, and people with lower educational attainment [27-29]. Lower digital literacy has also been found to be associated with lower educational attainment [30].

Data on ethnicity were collected due to the higher prevalence of low health literacy, and the concern about health inequalities, among BAME groups. Inequalities in health have been documented across ethnic groups in the United States and the United Kingdom, with Bangladeshi and Pakistani people reporting the poorest health, followed by Caribbean, Chinese, and Indian people [31,32]. White people have the best health [31,32]. Factors underlying these differences include SES, genetic, and cultural factors [33].

Considerable effort was invested during the development of HeLP-Diabetes to ensure that the program was accessible, relevant to, and met the needs of, people from a wide range of demographic backgrounds. The development of HeLP-Diabetes is described in more detail elsewhere [34]. It is a theoretically- informed, evidence-based online program developed using participatory design techniques and extensive user input, which has demonstrated efficacy in improving glycemic control [34,35].

The text for HeLP-Diabetes was written for people with a reading age of 12 (80% of UK population achieve this) [36]. All essential information was provided in a video as well as text, and personal stories were included, as people with low literacy prefer this method of learning [37-39].

HeLP-Diabetes was commissioned by 4 inner London clinical commissioning groups (CCGs) during the data collection period and offered to patients with T2DM as part of routine care. Hence there was a unique opportunity to gather real-world data on whether this intervention was being used across the digital divide. As the use of the program was a necessary prerequisite for patients obtaining health benefits [34], it was a relevant outcome for exploring whether the program was reaching the target audience.

### Aims

The overall aim of the study was to determine whether there was evidence of a digital divide when a Web-based self-management program for T2DM was integrated into routine care. Specific objectives were to determine:

1. Whether the demographic characteristics of people who registered to use the program differed from the target population, and if so how
2. Whether once registered, specific demographic groups were more likely to use the program
3. Whether there were different patterns of use by specified demographic characteristics

## Methods

### Design

A retrospective analysis of data on the use of HeLP-Diabetes in routine clinical practice in 4 inner London CCGs was undertaken.

### Setting

#### General Population of Study Setting

HeLP-Diabetes was commissioned by 4 inner London CCGs (CCG 1, 2, 3 and 4). All 4 CCGs have young, multicultural communities. They are densely populated and have relatively high levels of deprivation [40]. The educational attainment in the 4 CCGs is polarized. The proportion with degree level or above education attainment is higher than the national average. In contrast, 34%-42% of 19-year-old individuals do not have A-level qualifications (postsecondary nontertiary education) [41,42].

#### Diabetes Population of Study Setting

The target population of the HeLP-Diabetes program was adults with T2DM (see Table 1). There is a higher percentage of people in the 40-64 age group with T2DM in England (42.8%) and all 4 CGGs (range 49.3%-54.4%), than any other age group. More than 48% (range 48.3%-62.6%) of people with T2DM in the 4 participating CCGs are of BAME origin, reflecting the ethnic diversity of these areas [43].

### Intervention

HeLP-Diabetes is described in detail elsewhere [34]. It is an evidence-based, theoretically informed online self-management program for adults with T2DM. Content is based on the Corbin and Strauss [44] theory for living with long-term conditions which takes a holistic approach to diabetes management, incorporating the disease process (adopting healthy behaviors, working with health professionals, and taking medicines), the emotional consequences (the negative emotions associated with being diagnosed with a long-term condition), and the changes that occur in daily life (including the impact of a diagnosis on relationships with friends, family, and colleagues). Information is divided into 8 sections (see Table 2). Patients with T2DM were referred to the program by health care professionals, or made aware through flyers in waiting areas and texting from practices.

**Table 1:** Diabetes population of the clinical commissioning groups compared with England (prevalence is given as a percentage, because the numbers are not publicly available).

Population demographic characteristic			England	CCG^a^ 1	CCG 2	CCG 3	CCG 4
QoF^b^ total type 1 and type 2 diabetes prevalence, n (%)			3,116,399 (6.7)	15,213 (6.2)	10,368 (5.0)	18,274 (5.5)	16,663 (6.5)
**T2DM^c^ prevalence, (%)**							
	**Age (years)**						
		<40	3.9	4.6	4.8	4.9	4.8
		40-64	42.8	50.4	49.3	54.4	51.4
		65-79	38.0	32.8	32.2	29.2	31.0
		>80	13.8	10.0	10.6	9.6	11.0
	**Gender**						
		Male	55.8	51.9	52.5	52.5	51.7
		Female	48.1	44.2	47.5	47.5	48.3
	**Race**						
		White	64.4	33.8	49.2	31.3	41.4
		BAME^d^	19.3	60.5	48.3	62.6	55.8

^a^CCG: clinical commissioning groups.

^b^QoF: quality and outcomes framework in population >17 years of age.

^c^T2DM: type 2 diabetes mellitus.

^d^BAME: black and minority ethnic.

**Table 2:** Healthy Living for People with type 2 Diabetes (HeLP-Diabetes) website sections.

Section	Content
Understanding diabetes	Common diabetes questions How my body can be affected Quick guides
Staying healthy	Why is lifestyle important? Looking after yourself Physical activity Taking medicines Eating and drinking Alcohol Smoking Working with my diabetes team
Treating diabetes	How is type 2 diabetes treated? Tests to monitor diabetes Medicines Surgery Complimentary medicine Vaccinations and immunizations How the National Health Service can help
Living and working with diabetes	Food Relationships Work Social life Travel Driving Financial support Ramadan
Managing my feelings	Understanding my moods My mood tools
My health record	My diabetes care plan My appointments My health tracker My test results My medicines My reminders
News and research	News Research Concerns about specific medicines
Forum and help	Forum Useful resources People’s stories Frequently asked questions

The intervention was offered to patients with T2DM as a routine service in clinical practice. Practices placed flyers and posters in waiting areas informing patients about the program. Health care professionals were able to offer it to patients in consultations, and some practices wrote or sent texts to patients inviting them to register. Data were not recorded on how many patients were offered the program by health care professionals, and so this was not included in the analysis.

### Ethics and Privacy

Details of the people who used the HeLP-Diabetes website were automatically pseudoanonymized with a user identification. Pseudoanonymized data were collected by the server side of the website and subsequently exported by the research team to Microsoft Excel and then Tableau reader for analysis. Secondary analysis of information collected for service evaluation is excluded from an ethics committee review by the NHS Research Ethics Committees (RECs), as long as patients were not identifiable [45]. Formal ethical approval was therefore not needed.

### Data Collection

The demographic characteristics of everyone who registered to use the program were collected at the point of registration. Initially, people were registered and given access to the website by a member of the HeLP-Diabetes administrative team, and later a self-registration page was added to the website to allow people to register themselves. The demographic data collected at registration included gender, age, ethnicity, and education level. Education level was categorized using UK and US qualifications, and the International Standard Classification of Education [42].

The server side of the website automatically collected data on visits to the HeLP-Diabetes website. The data collected were: user ID, date and time of login, and page visited. These data were chosen as measures of use following best practice [46]. Alternative measures such as time can be prone to error as people may leave browsers on while engaged in alternative activities. The data presented here were collected between November 2015 and January 2017. During this time, 343 people registered to use the website, but not everyone who registered gave complete data on gender, ethnicity, education level and age. Therefore, the numbers provided in the results (n) are the numbers of people providing data for each demographic factor, and the totals are less than 343.

### Analysis

For the analysis, web page visits were grouped into 11 sections. Eight of these are the sections of the website (see Table 2), and the remaining 3 are other web pages that the user may have visited outside the 8 information sections of the HeLP-Diabetes website. These comprise of (1) the homepage, (2) miscellaneous articles, and (3) HeLP-Diabetes: Starting Out (a structured program for people newly diagnosed with T2DM based on the content of HeLP-Diabetes). The profile, administration, logout, and registration pages were excluded from the analysis. Statistical analysis was carried out using Stata (version 14.1) [47].

The analysis addressed each of the 3 research questions listed under the aims.

### Question 1: Did the Demographic Characteristics of People Who Registered to Use the Program Differ From the Target Population, and If so How?

The percentage of people who registered to use HeLP-Diabetes from each gender, ethnic, education, and age group was calculated to address this question. The target population was examined using the Public Health England data (see Table 1). A statistical analysis to compare the characteristics of the user population and the target population could not be carried out as the data were categorized differently. Instead of carrying out statistical analyses, we described the demographic characteristics of the registered users by stating the percentage of registered users in specified gender, ethnicity, education level and age groups. We have compared the percentage of male and female registered users with the percentage of males and females with T2DM in the 4 CCGs and compared the percentage of BAME registered users with the proportion of BAME people with T2DM in the 4 CCGs narratively. We were able to comment on which age group had the highest proportion of registered users.

### Question 2: Was There Evidence of the Digital Divide in Overall Use?

The term “use” was defined as logging in to the HeLP-Diabetes website at least twice. This was in order to determine who returned to the website, rather than just visiting once. The percentage of people who visited the website at least twice was calculated for each demographic group. Logistic regression analyses were performed to look for evidence of an association between the binary dependent variable (use/nonuse) and each of the covariates (gender, ethnicity, education level, and age group).

### Question 3: Was There Evidence of Differential Use by Demographic Characteristic?

The number of users who visited each of the 11 sections of the website and the number of visits to each section were categorized by demographic group. The Wilcoxon signed rank test was used to determine if there was an association between age and the number of visits to each section of the website. The Kruskal-Wallis equality of populations rank test was used to determine if there was an association between ethnicity, education, and the number of web page visits per user to each section of the website.

## Results

### Question 1: Was There Evidence of the Digital Divide in People Registered to Use the Program?

The mean age (see Table 3) was 58.4 years (SD 28.0). The age group with the highest proportion of people registered to use HeLP-Diabetes is the 51-60-year-olds (101/334, 30.2%), followed by the 61-70-year-olds (80/334, 24.0%), and 71-80-year-olds (56/334, 16.8%). Of the people with T2DM in the general population of the 4 CCGs (see Table 1), the highest proportion (range 49.3%-54.4%) of people was in the 40-64-year-old age group. This suggests that the age of the registered users reflected the target population. The most common education level was a bachelor’s degree or equivalent (102/299, 34.1%), followed by a general certificate of secondary education (GCSE)/high school diploma (89/299, 29.8%).

Males represented 55.5% (176/317) of registered users. Public Health data on the 4 CCGs the program was offered in, shows 51%-53% of people with T2DM in these areas are male. A total of 180/330 (54.5%) of the registered users were BAME, and 48%-60% of people with T2DM in the 4 CCGs are BAME. This suggested that the gender and ethnicity of people who registered to use the program reflected the target population in the 4 CCGs.

**Table 3:** Demographic characteristics of people who registered at clinical commissioning group 1 (n=97), 2 (n=51), 3 (n=154), and 4 (n=41) to use Healthy Living for People with type 2 Diabetes (HeLP-Diabetes).

Demographic characteristic		n (%)
**Gender (n=317)**		
	Male	176 (55.5)
	Female	141 (44.5)
**Ethnicity (n=330)**		
	White	150 (45.5)
	Black	117 (35.5)
	Asian	46 (13.9)
	Mixed	17 (5.2)
**Education level (n=299)**		
	GCSE^a^/high school	89 (29.8)
	A-level/postsecondary	64 (21.4)
	Bachelor’s degree or equivalent	102 (34.1)
	Master’s or doctoral degree or equivalent	44 (14.7)
**Age group (n=334)**		
	18-30	6 (1.7)
	31-40	20 (6.0)
	41-50	55 (16.5)
	51-60	101 (30.2)
	61-70	80 (24.0)
	71-80	56 (16.8)
	81-90	13 (3.9)
	≥91	3 (0.8)

^a^GCSE: general certificate of secondary education.

### Q2. Was There Evidence of the Digital Divide in Overall Use?“

Ninety-two of 150 (61.3%) white, 70/117 (59.8%) black, and 28/46 (60.9%) of Asians who registered to use HeLP-Diabetes, visited the website at least twice (see Table 4). This was lower in the mixed group (5/17 (29.4%), odds ratio (OR)=0.26, 95% CI=0.09-0.78). The median age of people who visited the website at least twice was 59 years (lower quartile=50, upper quartile=70), and age groups were categorized into quartiles for this analysis. Visits to the website by the different age groups ranged from 46.7% (50/107) in those aged 60-69 years to 55.9% (62/111) in those aged 51-59 years. There was no significant difference in usage for gender, education level or age.

### Q3. Were There Different Patterns of Use by Demographic Characteristics?

**Figure 1:**
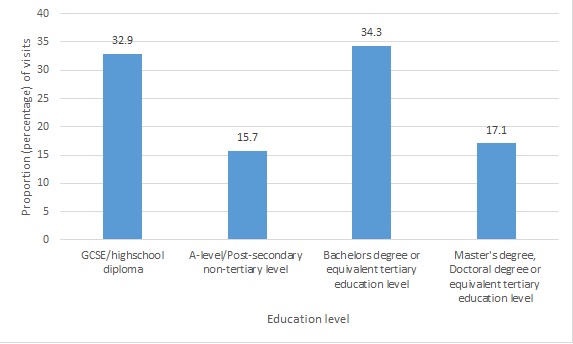
Proportion of visits to the "Living and working with diabetes" section of the website. GCSE: general certificate of secondary education.

**Figure 2:**
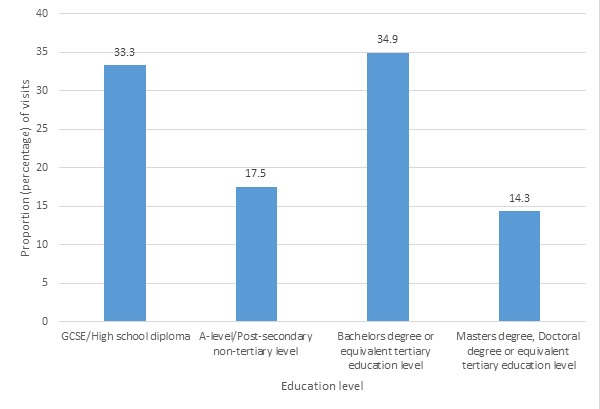
Proportion of visits to the "Treating Diabetes" section of the website. GCSE: general certificate of secondary education.

Overall, the 2 sections of the website that were most visited were (1) My health records and (2) Staying healthy. There was no evidence of differential use of the program by any demographic group, apart from education level, where there was weak evidence of an association between education level and visits to the “Living and working with diabetes” section with *P*=.03 (Figure 1), and the “Treating diabetes” section with *P*=.04 (Figure 2). The difference between visits by people with high school diplomas and a tertiary education level was small, as 34.3% (24/70) of visits to the “Living and working with diabetes” were by users with a bachelor’s degree or equivalent, while 32.9% (23/70) of visits were by users with high school diplomas. Also, 34.9% (22/63) of visits to the “Treating diabetes” section were by users with a bachelor’s degree or equivalent, compared to 33.3% (21/63) of visits by users with high school diplomas. The proportion of users who visited both sections with postsecondary nontertiary education level or master’s or doctoral degrees or equivalent was much lower (see Multimedia Appendices 1-4 for details).

**Table 4:** Proportion of people registered to use HeLP-Diabetes who visited at least twice.

Demographic characteristics		n/N^a^ (%)	Odds ratio (95% CI)	*P* value
**Gender (n=317)**				
	Female	77/141 (54.6)	1.00	.19
	Male	109/176 (61.9)	1.35 (0.86-2.12)	
**Ethnicity (n=335)**				
	White	92/150 (61.3)	1.00	.12
	Black	70/117 (59.8)	0.94 (0.57-1.54)	
	Asian	28/46 (60.9)	0.98 (0.50-1.93)	
	Mixed	5/17 (29.4)	0.26 (0.09-0.78)	
**Education level (n=299)**				
	GCSE^b^/high school	53/89 (59.6)	1.00	.95
	A-level/postsecondary	36/64 (56.3)	0.87 (0.46-1.67)	
	Bachelor’s degree or equivalent	61/102 (59.8)	1.06 (0.59-1.90)	
	Master’s degree, doctoral degree or equivalent	26/44 (59.1)	0.98 (0.47-2.05)	
**Age group (n=334)**				
	22-50	49/96 (51.0)	1.00	.54
	51-59	62/111 (55.9)	1.21 (0.70-2.10)	
	60-69	50/107 (46.7)	0.84 (0.49-1.46)	
	70-93	44/80 (55.0)	1.17 (0.65-2.13)	

^a^The proportion who visited the website at least twice (n)/everyone in this demographic group who registered (N).

^b^GCSE: general certificate of secondary education.

## Discussion

### Principal Results

This study is one of the first to explore whether there is evidence of a digital divide in the use of a digital health intervention integrated into routine health care. As such it makes a substantial addition to the literature on whether digital health is likely to increase or decrease health inequalities. Reassuringly, we found no strong evidence of differential patterns of registration, or patterns of use by age, gender, educational attainment or ethnicity. There was weak evidence that people from the mixed ethnicity group were less likely to use the program than the white group (OR=0.26, 95% CI=0.09-0.78, *P*=.12). There was also weak evidence of differences in visits to the “Living and working with diabetes” (*P*=.03) and “Treating diabetes” (*P*=.04) sections of the website by education level. The highest proportion of users who visited these sections of the website were those with a bachelor’s degree or equivalent, but people with high school diplomas closely followed.

### Comparison With Prior Work

These findings make a significant contribution because the literature on the use of digital health interventions suggests higher use among younger, well-educated, higher income, nonBAME individuals, which is a pattern that is likely to increase health inequalities. For example, a systematic review of electronic portal (an online electronic health records system) usage among patients with diabetes found that higher education, younger age, higher income, and nonHispanic, nonblack race were associated with higher portal utilization [48].

A second systematic review of patterns of user engagement with mobile and Web-based self-care interventions for adults with T2DM [49], also found that use was higher among younger people. However, 1 study included in the review showed that use of a mobile Health medication adherence promotion intervention for low-income adults with T2DM increased from 25 to 50 years of age, then decreased as age increased [50].

Our findings are also in keeping with a qualitative study of people with high and low levels of health literacy about a digital intervention to promote physical activity for diabetes in 5 countries [51]. Participants in that study were from areas with high levels of deprivation and had a mean age of 62 years and most found the design of the intervention was acceptable and engaging. Findings from both our study and this qualitative study suggest that it is possible to design digital health interventions that appeal to a diverse population, including people with low literacy and health literacy levels.

### Strengths and Limitations

A strength of the research is that individuals were offered HeLP-Diabetes as an NHS service, and not as a research study. This provides us with data on “real world” use of the program and not data generated from a highly controlled research setting. Actual website visits were automatically measured rather than using self-reported use of the program, which relies on memory and may result in bias from social desirability. A variety of engagement measures were analyzed including numbers who registered, the proportion who actively used the program, and the number of page visits. There are other measures of engagement including the duration of time spent using the website per visit, and the duration of time between visits to the website. The number of visits was considered to be a more reliable measure of engagement.

A limitation of the research is the total number of participants (n=343). This may limit the power of the study to detect significant differences between demographic groups. The 95% CI was provided in addition to *P* values in recognition of the fact that a *P* value by itself provides limited information [52]. Where the *P* value and 95% CI do not agree, this has been stated.

### Implications

The findings of this study suggest that digital health interventions can be designed to be used by people of different demographic backgrounds. This is important to enable equitable access to health information and support, and to prevent worsening health inequalities.

Developers of digital health interventions should be mindful of the needs of different demographic groups in their design process and involve users of different backgrounds at each stage of development. Research on the evaluation of digital health interventions should include the collection of data on the demographic profile of users, and the use (or other engagement measure) of the intervention by different demographic groups.

Developers also need to acknowledge and address barriers to the use of digital health interventions such as low health literacy and poor computer literacy. The 2014 Government Digital Inclusion Strategy has identified lack of computer skills, not having the motivation to go online, and lack of trust in internet security as additional challenges to internet use, in addition to lack of access [19]. Possible reasons for our relative success in crossing the digital divide are twofold. First, full integration into routine health care, with a recommendation for use from health care professionals. This improved motivation to go online and use the intervention, and trust in the security of the intervention [53]. Second, careful design of the intervention to make it fully accessible to a wide range of people. HeLP-Diabetes was designed using participatory design techniques, extensive user input, and consideration of literacy levels and use of audio-visual media [34]. These techniques make the program more accessible to people with lower health literacy and computer literacy.

### Conclusion

This study is one of the first to provide evidence that health inequalities are not necessarily widened when a digital health intervention was integrated into routine health care. Weak evidence of a difference in overall use was identified for ethnicity (less use by the mixed-race ethnic group). here was also weak evidence of differences in the use of the “Living and working with diabetes” and “Treating diabetes” sections of the website (the highest proportion of visits were by people with a bachelor’s degree) but the proportion of visits by people with high school diplomas was very similar). The relative success of the intervention may be attributed to integration into routine health care, and recommendation from health care professionals, but also careful design with extensive user input and consideration of literacy levels. Developers of digital health interventions need to acknowledge barriers to access and use including health literacy, computer literacy, motivation and concerns about internet security if they are to navigate and reduce health inequalities successfully.

## Appendix

Multimedia Appendix 1 Total number of visits to each section of the HeLP-Diabetes website by female and male users.

Multimedia Appendix 2 Total number of visits to each section of the HeLP-Diabetes website by users of different education levels.

Multimedia Appendix 3 Total number of visits to each section of the HeLP-Diabetes website by users of different age groups.

Multimedia Appendix 4 Total number of visits to each section of the HeLP-Diabetes website by users of different ethnic groups.

## Abbreviations

BAME: black and minority ethnic
CCG: clinical commissioning group
GCSE: general certificate of secondary education
HeLP-Diabetes: Healthy Living for People with type 2 Diabetes
NHS: National Health Service
SES: socioeconomic status
T2DM: type 2 diabetes mellitus
