# Supplementary material for: Crossing the Digital Divide in Online Self-Management Support: Analysis of Usage Data From HeLP-Diabetes
Source: JMIR Diabetes. 2018 Dec 6;3(4):e10925. doi: 10.2196/10925 (PMC6303008; doi:10.2196/10925)
Supplement: Multimedia Appendix 1 [file diabetes_v3i4e10925_app1.pdf]

Appendix 1

Total number of visits to each section of the HeLP-Diabetes website by female and male users

**Website section**	**Total number of visits by female users**	**Total number of visits by male users**	***p***
Forum and help	157	343	0.50
Homepage	417	536	0.29
Living and working with diabetes	118	306	0.48
Managing my feelings	104	132	0.89
Miscellaneous articles	83	110	0.33
My health records	505	767	0.81
News and research	43	93	0.21
Profile, admin, login, logout or register pages	404	580	0.20
HeLP-Diabetes: Starting Out	362	525	0.56
Staying Healthy	531	610	0.56
Treating Diabetes	104	239	0.07
Understanding Diabetes	370	434	0.54
