# Supplementary material for: Crossing the Digital Divide in Online Self-Management Support: Analysis of Usage Data From HeLP-Diabetes
Source: JMIR Diabetes. 2018 Dec 6;3(4):e10925. doi: 10.2196/10925 (PMC6303008; doi:10.2196/10925)
Supplement: Multimedia Appendix 2 [file diabetes_v3i4e10925_app2.pdf]

Appendix 2

Total number of visits to each section of the HeLP-Diabetes website by users of different education levels

**Website section**	**School leaver**	**A-level**	**Degree or NVQ4**	**Post Grad or NVQ5**	***p***
Forum and help	233	52	135	66	0.30
Homepage	287	188	283	144	0.96
Living and working with diabetes	193	92	106	27	0.03
Managing my feelings	61	45	82	43	0.22
Miscellaneous articles	45	47	77	24	0.95
My health records	256	287	428	279	0.54
News and research	50	22	24	41	0.59
Profile, admin, login, logout or register pages	350	178	285	122	0.79
HeLP-Diabetes: Starting Out	248	165	341	125	0.71
Staying Healthy	466	201	297	154	0.32
Treating Diabetes	115	30	129	71	0.04
Understanding Diabetes	251	225	234	67	0.93
