# Supplementary material for: Crossing the Digital Divide in Online Self-Management Support: Analysis of Usage Data From HeLP-Diabetes
Source: JMIR Diabetes. 2018 Dec 6;3(4):e10925. doi: 10.2196/10925 (PMC6303008; doi:10.2196/10925)
Supplement: Multimedia Appendix 3 [file diabetes_v3i4e10925_app3.pdf]

Appendix 3

Total number of visits to each section of the HeLP-Diabetes website by users of different age groups

**Website section**	**18-30**	**30-40**	**40-50**	**50-60**	**60-70**	**70-80**	**80-90**	**90 and over**	***p***
Forum and help	14	28	27	94	202	94	61	0	0.24
Homepage	3	59	151	347	249	125	45	9	0.21
Living and working with diabetes	4	27	49	186	127	28	17	6	0.18
Managing my feelings	11	15	23	75	64	49	5	0	0.63
Miscellaneous articles	3	11	27	77	57	34	5	0	0.94
My health records	46	133	89	433	377	180	43	4	0.50
News and research	0	4	2	36	52	26	17	0	0.21
Profile, admin, login, logout or register pages	9	46	120	287	275	208	75	8	0.76
HeLP-Diabetes: Starting Out	3	49	66	282	283	207	42	3	0.98
Staying Healthy	16	47	80	408	290	260	73	10	0.85
Treating Diabetes	0	25	33	116	130	31	11	10	0.48
Understanding Diabetes	2	31	92	351	134	141	61	19	0.70
