# Supplementary material for: Crossing the Digital Divide in Online Self-Management Support: Analysis of Usage Data From HeLP-Diabetes
Source: JMIR Diabetes. 2018 Dec 6;3(4):e10925. doi: 10.2196/10925 (PMC6303008; doi:10.2196/10925)
Supplement: Multimedia Appendix 4 [file diabetes_v3i4e10925_app4.pdf]

Appendix 4

Total number of visits to each section of the HeLP-Diabetes website by users of different ethnic groups

**Web page**	**White**	**Black**	**Asian**	**Mixed**	***p***
Forum and help	42	29	17		0.12
Homepage	83	64	27	4	0.45
Living and working with diabetes	37	24	12	1	0.89
Managing my feelings	35	21	11	1	0.12
Miscellaneous articles	48	32	13	2	0.80
My health records	45	29	15	1	0.70
News and research	16	10	4	0	0.16
Profile, admin, login, logout or register pages	100	76	31	6	0.19
HeLP-Diabetes: Starting Out	64	38	16	2	0.66
Staying Healthy	77	55	21	3	0.86
Treating Diabetes	36	25	4	0	0.22
Understanding Diabetes	42	35	18	2	0.18
